# Transcatheter closure of atrial septal defect in adults: time-course of atrial and ventricular remodeling and effects on exercise capacity

**DOI:** 10.1007/s10554-019-01647-0

**Published:** 2019-06-15

**Authors:** Sigurdur S. Stephensen, Ellen Ostenfeld, Shelby Kutty, Katarina Steding-Ehrenborg, Hakan Arheden, Ulf Thilén, Marcus Carlsson

**Affiliations:** 1 Department of Clinical Sciences, Clinical Physiology, Lund University, Skåne University Hospital, 22241 Lund, Sweden; 2 Department of Clinical Sciences, Pediatric Cardiology, Lund University, Skåne University Hospital, Lund, Sweden; 3 Helen B Taussig Heart Center, Johns Hopkins Hospital and School of Medicine, Baltimore, MD USA; 4 Department of Health Sciences, Physiotherapy, Lund University, Lund, Sweden; 5 Department of Clinical Sciences, Cardiology, Lund University, Skåne University Hospital, Lund, Sweden

**Keywords:** Heart failure, Exercise capacity, Congenital heart disease, ASD, MRI

## Abstract

Investigate ventricular and atrial remodeling following atrial septal defect (ASD) closure and examine if pulmonary-to-systemic flow ratio (QP/QS) and right ventricular (RV) volume predict improvement, determined as percentage of predicted oxygen uptake (VO_2_%). Long-term cardiovascular magnetic resonance (CMR) data on atrial and ventricular remodeling after ASD-closure is limited and treatment effect on exercise capacity is debated. Sixteen patients undergoing transcatheter ASD closure and 16 age and sexmatched controls were studied. CMR was performed before treatment, the day after and 3 and 12 months later. Exercise test with gas analysis was performed before and 12 months after treatment. QP/QS decreased from 2.1 ± 0.5 to 1.4 ± 0.3 at day 1 and 1.1 ± 0.1 at 3 and 12 months. Left ventricular (LV) volumes increased and normalized on day 1 whereas left atrial volumes were unchanged. RV and right atrial volumes decreased the first 3 months. LV and RV volumes had not equalized at 12 months (RV/LV ratio 1.2 ± 0.1, P < 0.01) and RV ejection fraction remained decreased compared to controls. Improvement of VO_2_% after ASD closure (P < 0.01) was inversely related to QP/QS at rest (r = − 0.56, P < 0.05) but unrelated to RV end-diastolic volume (P = 0.16). Following transcatheter ASD closure, LV adaptation is rapid and RV adaptation is prolonged, with decreased systolic RV function. Patients with smaller shunts had larger improvement in VO_2_% suggesting patients with defects of borderline hemodynamic significance might benefit from closure. This may be due to impaired LV diastolic function influencing shunt size and exercise capacity following ASD closure.

## Introduction

The indications for surgical or transcatheter closure of secundum atrial septal defects (ASD) are enlargement of the right ventricle (RV) or symptoms including exercise intolerance, fatigue or dyspnea [1]. The long term outcome after ASD closure is good, but risk of arrhythmia with concomitant neurological events remains [2]. Limited data on atrial and ventricular remodeling after ASD closure is available from echocardiography [3] but more comprehensive long-term follow-up information ( > 6 months) on left and right atrial and ventricular volumes with CMR is unavailable. Knowledge of cardiac chamber remodeling is important for understanding the results of treatment and when evaluating patients after ASD closure. Studies with CMR have found residual RV enlargement compared to the left ventricle (LV) at 6 months [4]. Schoen et al. found 30% of patients to have enlarged RV at 12 month follow-up [5]. However, the long-term adaptation of both ventricles and atria following ASD closure is unknown. Decreased exercise tolerance in ASD patients has been related to decreased LV filling and stroke volume [6], increased pulmonary artery pressure during exercise [7] and altered interventricular interaction [8] related to the volume overload of the RV and abnormal septal motion. Even though many patients subjectively report receding dyspnea and improved exercise tolerance after ASD closure, objective data does not always support this. Several studies have reported the short- and long-term effects of ASD closure on patients and many of them demonstrate improved cardiopulmonary function [8–12], even in patients with a relatively small shunt ratio [13]. However, other studies have either shown no improvement in exercise capacity following defect closure [14, 15] or impaired exercise response compared to healthy controls [16, 17]. Therefore, better understanding of which patients will benefit from closure of ASD is of value.

The aim of the present study was to show the time course of ventricular and atrial remodeling following transcatheter closure of secundum ASD and determine if pulmonary-to-systemic flow ratio (QP/QS) and RV volume could predict improvement after treatment, as determined by predicted peak oxygen uptake (VO_2_%).

## Methods

### Study population and design

The study was approved by the Regional Ethical Review Board in Lund, Sweden and written informed consent was obtained from all patients. Sixteen patients (11 females) with hemodynamically significant secundum ASD (QP/QS > 1.5) scheduled for transcatheter ASD closure were included and underwent CMR and exercise testing with direct respiratory gas analysis. All patients willing to participate in the study were included, except for patients with atrial fibrillation. CMR was repeated in patients the day after transcatheter closure of the defect and 3 months and 12 months after treatment. Exercise test with respiratory gas analysis was repeated at 12 months follow-up. Sixteen age and sex-matched controls were also examined with CMR and exercise test with 12 months’ time interval.

### CMR imaging

All subjects were planned for CMR at rest in supine position and during end-expiratory breath hold. A 1.5 T CMR scanner was used for all studies (Philips Achieva, Best, The Netherlands). Steady state free precession cine CMR images were acquired in three long-axis planes and in short-axis stacks covering the whole heart. Imaging parameters for cine CMR were typically: retrospective ECG triggering with acquired temporal resolution of 47 ms reconstructed to 30 time phases per cardiac cycle, repetition time 3 ms, echo time 1.4 ms, flip angle 60°, slice thickness of 8 mm with no slice gap. Breath-holds were typically 10 s. Flow velocity mapping was acquired using a retrospectively ECG triggered fast-field echo velocity encoded sequence, acquired as separate acquisitions in the ascending aorta and the pulmonary trunk during free breathing. Imaging parameters were typically: repetition time 10 ms, echo time 5 ms, flip angle 15°, and slice thickness 8 mm, acquired in-plane resolution 2.4 × 2.4 mm reconstructed to 1.3 × 1.3 mm, number of acquisitions 1, no parallel imaging and a velocity encoding gradient (VENC) of 200 cm/s. The flow sequence had an acquired temporal resolution of 20 ms during the cardiac cycle reconstructed to 35 phases per heart cycle and a typical scan time of 2 min.

### Image analysis

All image analysis was performed using Segment, v1.9 (https://segment.heiberg.se) [18]. Ventricular end-diastolic (ED) and end-systolic (ES) volumes (EDV and ESV) and stroke volumes (SV) were obtained by delineating the endocardial borders of the left ventricle (LV) and right ventricle (RV) in all slices of the short-axis stack. The maximum left and right atrial volume (LAV and RAV) were delineated in contiguous short axis slices through the entire atria during ventricular end-systole. Appendages were included, and veins were excluded from LAV and RAV. All atrial and ventricular volumes were indexed (i) to body surface area (BSA). Flow images were used to calculate cardiac output (CO) in the aorta and pulmonary trunk [19] and the shunt ratio between pulmonary and aortic flow (QP/QS) was calculated. The shunt volume per heart beat was calculated from the difference in flow per heart beat between the pulmonary trunk and aorta.

### Exercise test with continuous gas analysis

Exercise testing was performed before ASD closure and 12 months later, using Monark 939E cycle ergometer and Oxygen Pro (Jaeger, Hochberg, Germany). Peak oxygen uptake (VO_2_*peak*) was defined as the average of three highest VO_2_ values documented during the last minute of the test. Blood pressure and a 12 lead ECG were monitored during exercise. VO_2_*peak* percent of predicted value (VO_2_%) was calculated according to the Hansen/Wasserman equation [20].

### Statistical analysis

All statistical analysis was performed using Graphpad Prism v 7.0. Continuous variables are presented as mean ± SD and categorical variables in absolute numbers and percent. Pearson’s correlation was used to examine relationship between left-to-right shunting or ventricular volume and results from the exercise test. Mann–Whitney and Wilcoxon tests were used to test if variables differed among the groups. Results with a two-sided P-value of less than 0.05 were considered statistically significant. Inter-observer variability was calculated as bias ± SD according to Bland–Altman in 9 ASD patients and 6 healthy controls for LVSV and RVSV.

## Results

### Patient characteristics

Subject characteristics and clinical function according to the New York Heart Association scale are presented in Table 1. The ASD devices used for closure were Amplatzer Septal Occluder (St. Jude Medical - Abbott, St. Paul, Minneapolis, MN). Controls were matched for age and sex and had similar BSA and heart rate compared to patients at 12 months follow up (Table 1). One patient declined the 12 months follow-up visit, and one patient declined the repeated exercise test. New York Heart Association (NYHA) functional class improved at 12 months follow-up with more patients in NYHA class I (87% vs. 38%, p < 0.05) after intervention.

**Table 1:** Comparison of baseline characteristics between patients and controls

	Preop (n = 16)	Postop, day 1 (n = 16)	3 months follow-up (n = 16)	12 months follow-up (n = 15)	Controls (n = 16)
Age (years)	52 ± 17	52 ± 17	53 ± 17	53 ± 18	46 ± 12
Females n (%)	11 (69%)	11 (69%)	11 (69%)	10 (63)	11 (69%)
BSA (m^2^)	1.8 ± 0.2			1.9 ± 0.2	1.8 ± 0.2
VO_2_ peak (mL/kg/min)	26 ± 7			28 ± 8	42 ± 10***
VO_2_ peak (%)	101 ± 19			108 ± 19^§§^	152 ± 27***
Heart rate (bpm)	71 ± 10	63 ± 6^††^	62 ± 12	63 ± 9	64 ± 8
NYHA class	I = 6 (38%), II = 7 (43%), III = 3 (19%)			I = 13 (87%), II = 2 (13%)^§^	
Mean PAP (mm Hg)	19 ± 5				
Mean LAP (mmHg)	7 ± 3				

*Bpm* beats per minute, *BSA* body surface area, *VO*_*2*_*peak* peak oxygen uptake, *NYHA* New York Heart Association, *PAP* pulmonary artery pressure, *LAP* left atrial pressure

***P < 0.001 when comparing preop and controls

^§^P < 0.05

^§§^P < 0.01 when comparing preop and 12 months follow-up

^††^P < 0.01 when comparing preop and postop, day 1

### Time course of ventricular and atrial remodeling after ASD-closure

**Fig. 1:**
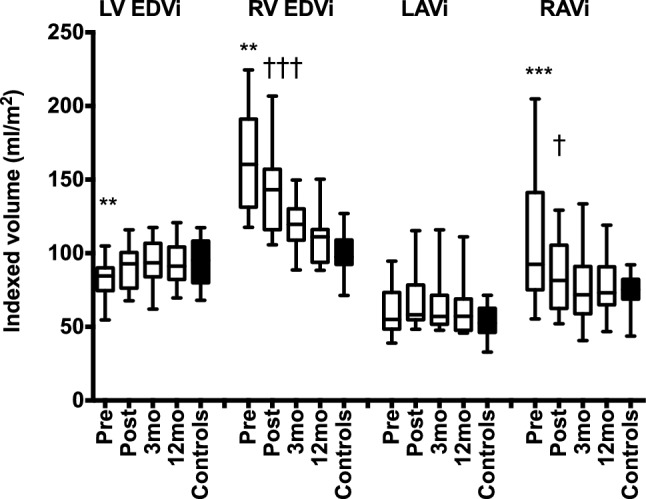
**Ventricular and atrial volumes at serial CMR studies.** Ventricular and atrial remodeling over 1 year after transcatheter closure of atrial septal defects (ASD) compared to controls. All volumes are indexed (i) to body surface area. Left ventricular (LV) end diastolic volume (EDVi) increased the day after ASD-closure. Right ventricular (RV) EDVi and right atrial maximum volume (RAVi) decreased the day after and further decreased 3 months (3 mo) after closure but RV EDVi was still larger compared to LV EDVi at 12 months (12 mo) (P < 0.01). Left atrial maximum volume (LAVi) did not change after closure. **P < 0.01 pre versus post ASD-closure, ^†^P < 0.05 post versus 3 mo, ^†††^P < 0.001 post versus 3 mo

The QP/QS was 2.1 ± 0.5 before intervention (Table 2). There was a correlation between RV size and QP/QS (r = 0.87, P < 0.001 for RVEDVi and r = 0.70, P < 0.005 for RVESVi). There was a small residual shunt on day 1 (1.4 ± 0.3), that was not present at 3 or 12 months (1.1 ± 0.1, P < 0.001, Table 2). The LVEDVi and LVESVi volumes had already increased on day 1 after ASD closure (P < 0.01 for both) and remained unchanged over the next 12 months (Fig. 1, Table 2). There was no change in LVSVi from before closure to 12 months follow-up (P = 0.057, Table 2). The LVEDVi in patients at 12 months was similar to that of controls. RVEDVi decreased the day after intervention (P < 0.001) and ventricular remodeling continued with further decrease in RVEDVi at 3 months (P < 0.001) but was thereafter unchanged at 12 months (Fig. 1). RVESVi was stable from pre- to post-intervention (P = 0.14), decreased at 3 months follow-up after ASD closure (P < 0.001), but remained larger than in controls at 12 months (P < 0.01). RVSVi decreased on the first day after closure (P < 0.05) and remained unchanged at 3 and 12 months. The global systolic function quantified as ejection fraction (EF) remained decreased compared to controls at 12 months follow up. The RV volumes were still larger than LV volumes (P < 0.01 for both RVEDVi and RVESVi) but RVSVi did not differ (P = 0.39).

**Table 2:** Atrial and ventricular volumes, function and flow measurements

	Preop (n = 16)	Postop, day 1 (n = 16)	3 months follow-up (n = 16)	12 months follow-up (n = 15)	Controls (n = 16)
LVEDVi (mL/m^2^)	83 ± 13	91 ± 14**	93 ± 16	93 ± 14	94 ± 16
LVESVi (mL/m^2^)	36 ± 11	40 ± 12**	43 ± 10	41 ± 11	40 ± 11
LVSVi (mL/m^2^)	47 ± 6	51 ± 8	51 ± 7	51 ± 7	53 ± 9
LVEF (%)	58 ± 8	57 ± 8	55 ± 5	57 ± 7	57 ± 7
RVEDVi (mL/m^2^)	171 ± 53	145 ± 31***	118 ± 16^†††^	110 ± 16	100 ± 16
RVESVi (mL/m^2^)	82 ± 27	79 ± 23	64 ± 15^††^	57 ± 10	44 ± 7^§§^
RVSVi (mL/m^2^)	89 ± 30	66 ± 14**	59 ± 14	53 ± 10	51 ± 15
RVEF (%)	52 ± 6	46 ± 7**	48 ± 6	48 ± 5	56 ± 4^§§^
LAVi (mL/m^2^)	61 ± 17	66 ± 19	66 ± 20	63 ± 21	54 ± 11
RAVi (mL/m^2^)	103 ± 42	88 ± 26***	78 ± 27^†^	76 ± 19	74 ± 12
Systemic CI (L/min/m^2^)	2.8 ± 0.4	2.5 ± 0.7	2.7 ± 0.6	2.9 ± 0.5	3.4 ± 0.5
Pulmonary CI (L/min/m^2^)	5.6 ± 0.9	3.6 ± 0.7***	3.0 ± 0.6^†^	3.0 ± 0.5	3.5 ± 0.6
QP/QS	2.1 ± 0.5	1.4 ± 0.3***	1.1 ± 0.1^††^	1.1 ± 0.1	1.0 ± 0.1

*LVEDVi* left ventricular end diastolic volume indexed to body surface area (BSA), *LVESVi* left ventricular end systolic volume indexed to BSA, *LVSVi* left ventricular stroke volume indexed to BSA, *LVEF* left ventricular ejection fraction, *RVEDVi* right ventricular end diastolic volume indexed to BSA, *RVESVi* right ventricular end systolic volume indexed to BSA, *RVSVi* right ventricular stroke volume indexed to BSA, *RVEF* right ventricular ejection fraction, *LAVi* left atrial volume indexed to BSA, *RAVi* right atrial volume indexed to BSA, *CI* cardiac index, *QP/QS* pulmonary-to-systemic flow ratio

**P < 0.01, ***P < 0.001 when comparing preop and postop day one

^†^P < 0.05, ^††^P < 0.01, ^†††^P < 0.001 when comparing postop day one and 3 months follow-up

^§§^P < 0.01 when comparing 12 months follow up to controls

**Fig. 2:**
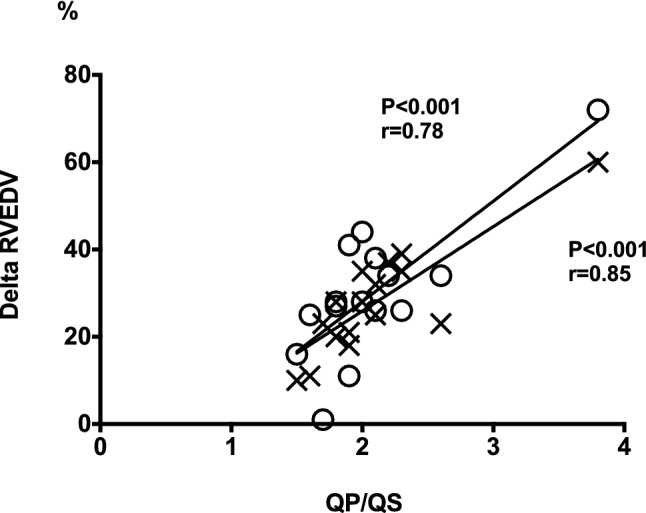
**Relationship between left-to-right shunting and change in right ventricular volume.** Linear correlation between shunt size (QP/QS) prior to ASD closure and the relative change in right ventricular end-diastolic volume (RVEDV) 3 months (crosses) and 12 months (circles) after ASD closure

The LAVi did not change after ASD closure. The numerically larger LAVi in patients at 12 months compared to controls was not statistically significant (P = 0.44). However, RAVi decreased the day after closure with further decrease at 3 months. At 12 months, RAVi did not differ between patients and controls. The QP/QS (r = 0.79) and shunt per heartbeat (r = 0.83) correlated with the RVEDV/LVEDV ratio before ASD closure (P < 0.001 for both) and the change in RVEDV from baseline to 3 and 12 months follow up had a strong correlation with shunt size prior to ASD closure (Fig. 2). The interobserver variability was 6 ± 4% for LVSV and 1 ± 3% for RVSV.

### Peak oxygen uptake

**Fig. 3:**
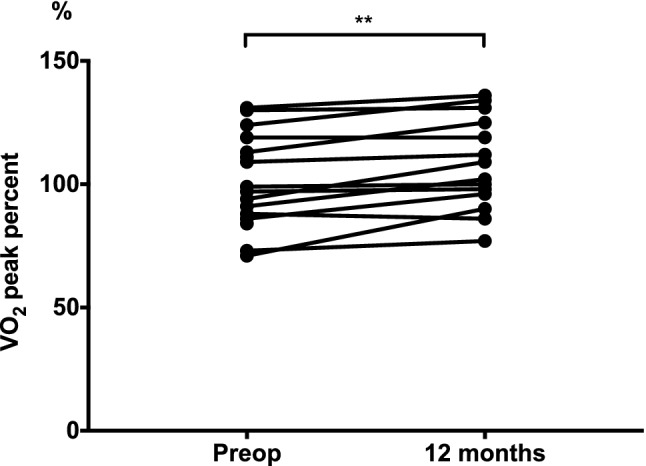
**Peak oxygen uptake before and after ASD closure.** Peak oxygen uptake as percentage of predicted value (VO_2_%) on exercise test before ASD closure and 12 months later

**Fig. 4:**
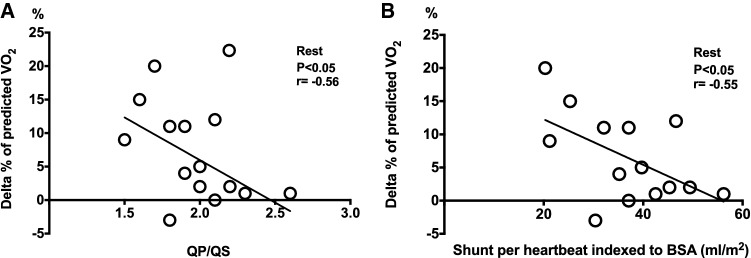
**Relationship between left-to-right shunting and change in peak oxygen uptake with treatment.** The correlation between pulmonary-to-systemic flow ratio (QP/QS) before closure (**a**) and the change in predicted peak oxygen uptake (VO_2_%) 12 months after ASD-closure and the shunt per heart beat indexed to body surface area (BSA) before closure (**b**) and the change in predicted VO_2_%. Smaller shunt size had a moderate correlation with improved exercise capacity

Absolute peak oxygen uptake (VO_2_*peak*) from cardiopulmonary exercise testing did not differ before ASD closure (25.6 ± 7.2 mL/kg/min) and 12 months later (27.6 ± 8.1 mL/kg/min, P = 0.07), but the VO_2_ as percent of predicted value (VO_2_%) improved from 101 ± 19% before to 108 ± 19% at 12 months (P < 0.01, Fig. 3). Both absolute and predicted VO_2_ were lower before closure and at 12 months compared to controls (P < 0.001, Table 2). There was a moderate inverse correlation (r = − 0.56, p < 0.05) between the shunt size at rest and improvement in VO_2_% at 12 months compared to pre-operative VO_2_%. Thus, patients with larger QP/QS at rest prior to ASD closure and patients with larger left-to-right shunt per heart beat indexed to BSA had lower improvement in VO_2_% at 12 months follow-up (Y = − 0.024 × X + 2.12 for QP/QS and Y = − 0.871 × X + 42.63 for shunt per heartbeat, Fig. 4). Improvement in VO_2_% did not correlate with RV size at rest (P = 0.16 for RVEDVi and P = 0.40 for RVESVi), change in RV size from baseline to 12 months follow-up (P = 0.28 for RVEDVi and P = 0.66 for RVESVi), or with RVEDV/LVEDV ratio before ASD closure (P = 0.54). There was no correlation between improved VO_2_% and the patients’ age (P = 0.77).

## Discussion

This study is the first to longitudinally investigate secundum ASD patients treated with catheter mediated device closure with serial CMR examinations up to 1 year after closure. Four-chamber volumetric evaluation showed differences in cardiac adaptation between the left and right ventricles. The LV adaptation was rapid, as early as the first day after closure of the ASD, whereas the changes in RV volumes were more gradual and plateaued at 3 months. Right atrial volumes decreased till 3 months, but left atrial volumes did not change after ASD-closure. Patients had similar LV and LA volumes and global systolic LV function after treatment compared to controls, even though the exercise capacity was much lower in the former. The enlarged RV end-systolic and reduced RV systolic function at 12 months after closure may explain this, or alternatively this may be related to impaired LV diastolic function. We observed the improvement of predicted oxygen uptake (VO_2_%) at 12 months follow up to be lower in adult ASD patients with larger shunts compared to smaller shunts. This indicates that patients who have had increased pulmonary blood flow for a long time may not have the same positive outcome of treatment as expected in children or younger aged patients [21]. A possible explanation for this finding is that ASD patients with impaired LV diastolic function have a larger left-to-right shunt. We speculate that the long-term LV diastolic dysfunction may limit the potential for improved peak oxygen uptake after ASD closure. Another possible explanation may be that patients with larger shunts need longer follow up times to demonstrate improvements in exercise capacity.

### Ventricular and atrial adaptation

The RVEDV and RVESV responded in the same manner to decreased volume-load, with a steady state reached at 3 months after ASD closure. Our results on atrial and ventricular adaptation to decreased volume-load are in concordance with other studies on ASD patients [3–5, 22]. However, we found a residual larger RV compared to LV at 12 months not reported in the studies using echocardiography. This is likely explained by methodological differences, where evaluation of RV volumes by echocardiography is challenging and even with 3D-echocardiography RV volumes are underestimated compared to CMR [23]. Our results show slightly larger RV volumes at follow up compared to Schoen et al. [5] and this may be due to the relatively older patients, larger RV volumes before closure and higher degree of patients in NYHA II and III (62%) in our study. Teo et al. [4] showed similar RV/LV EDV ratio at 6 months (1.3:1) and the present study extends this finding to 12 months.

### Oxygen uptake

The improvement in oxygen uptake for absolute values from before closure to 12 months follow-up did not reach statistical significance, while VO_2_*peak* as percentage of predicted value (VO_2_%) did. The lack of increase in absolute VO_2_*peak* does not translate to absence of subjectively experienced improvement. Quality of life has been shown to improve after ASD closure [24] and indeed NYHA class improved in our population. Our cohort of patients unexpectedly had normal exercise capacity before ASD closure (VO_2_% = 101%) whereas others have reported considerably lower values [9, 12, 13]. Our control group had much greater exercise capacity (VO_2_% = 152%). The pretreatment normal VO_2_*peak* in our patient cohort could explain the unchanged absolute VO_2_*peak* at follow up, as they may have less potential for improvement compared to patients with impaired pretreatment exercise capacity. Previous studies have shown conflicting results after ASD closure with both improved and impaired exercise capacity reported [8, 13, 17]. Giardini et al. found a correlation between improved *VO*_*2*_*peak* and LVEF [8]. LVEF did not change with ASD closure in our population and RVEF was unchanged at follow-up whereas others have shown increased RVEF 6 months after ASD closure [4, 5]. The potential for improved RVEF is likely related to the degree of pretreatment RV dysfunction, since RVEF in these previous studies were considerably lower than in our study. Another possible reason for the relatively small improvement in *VO*_*2*_*peak* is that patients may have adapted to a certain degree of physical limitation even if NYHA class improved. Indeed, a study by Kröönström et al. found that adults with congenital heart defects have impaired isotonic muscle function compared to healthy subjects [25]. Low isotonic muscle function is considered a marker for a generally sedentary lifestyle in these patients [25]. Therefore, undergoing treatment of the heart defect may not, on a short-term basis, be expected to change lifestyle. Giardini et al. found increased predicted oxygen uptake at > 36 months after treatment and the increase correlated with the degree of pretreatment shunt [9]. It appears that patients with larger shunts need longer time to adapt to the new physiology and this may explain the inverse relation between shunt size and increased predicted exercise capacity at 12 months follow up.

Diastolic dysfunction that becomes unmasked with ASD closure could be a third factor explaining the modest improvement in exercise capacity in our patients. Increased LV end-diastolic pressure has been reported following ASD closure in the older population, where abnormal LV relaxation, increased LV stiffening and impaired contraction due to chronic underload may coexist [26–28]. In our cohort, the LA pressure was not elevated before closure (Table 1), but we do not have LA or LV pressure data after closure. We speculate the smaller improvement in VO_2_% in patients with larger shunts to be related to LV diastolic function and shunt size because a patient with large ASD and impaired LV diastolic function will have a large shunt and a patient with large ASD and normal LV diastolic function will have a smaller shunt. In addition, patients with diastolic dysfunction may also have increased LV filling pressure during exercise - another cause for reduced pretreatment exercise capacity. This relationship is highlighted in the recent clinical trials of using interatrial shunt device to treat patients with LV diastolic dysfunction and increased LV filling pressure [29]. LA volumes are generally increased with elevated filling pressures [30], but that was not seen in this cohort.

### Limitations

The sample size is small which is explained by this being a single center study with a relatively small population. Although we aimed to include age matched controls, there were still a difference in age between healthy controls and patients (mean age 46 vs. 52 years). This could potentially affect cardiac volume comparisons as they are age related, but this effect is probably small. The influence on VO_2_ max is probably negligible as we used age-predicted values. Invasive pressure data after closure of the ASD was not available but would have been helpful to determine if diastolic dysfunction explains the limited improvement in exercise capacity. MRI data on transmitral flow was only obtained from a few patients and thus was not sufficient for assessment of LV diastolic function.

## Conclusion

The time course of ventricular remodeling after ASD closure is faster in the LV compared to the RV. Enlarged RV volume remained in our cohort after one year. The RA volumes showed reverse remodeling until 3 months but LA volumes did not change at all. Exercise capacity was lower in patients even with no difference in LV volumes and function from controls, which may be related to LV diastolic dysfunction or reduced RV global function. The improvement in peak oxygen uptake after ASD closure in adult patients is not straightforward. Patients with smaller shunts in this series had larger improvement in predicted exercise capacity, which suggests that patients with small ASDs also might benefit from closure.
